# Ectoparasitism shortens the breeding season in a colonial bird

**DOI:** 10.1098/rsos.140508

**Published:** 2015-02-18

**Authors:** Charles R. Brown, Mary Bomberger Brown

**Affiliations:** 1 Department of Biological Sciences, University of Tulsa, 800 South Tucker Drive, Tulsa, OK 74104, USA; 2 School of Natural Resources, University of Nebraska, Lincoln, NE 68583, USA

**Keywords:** cliff swallow, ectoparasites, *Petrochelidon pyrrhonota*, reproductive phenology, swallow bug, time of breeding

## Abstract

When blood-feeding parasites increase seasonally, their deleterious effects may prevent some host species, especially those living in large groups where parasites are numerous, from reproducing later in the summer. Yet the role of parasites in regulating the length of a host's breeding season—and thus the host's opportunity for multiple brooding—has not been systematically investigated. The highly colonial cliff swallow (*Petrochelidon pyrrhonota*), a temperate-latitude migratory songbird in the western Great Plains, USA, typically has a relatively short (eight to nine week) breeding season, with birds rarely nesting late in the summer. Colonies at which ectoparasitic swallow bugs (*Oeciacus vicarius*) were experimentally removed by fumigation were over 45 times more likely to have birds undertake a second round of nesting than were colonies exposed to parasites. Late nesting approximately doubled the length of the breeding season, with some birds raising two broods. Over a 27 year period the percentage of birds engaging in late nesting each year increased at a colony site where parasites were removed annually. This trend could not be explained by changes in group size, climate or nesting phenology during the study. The results suggest that ectoparasitism shortens the cliff swallow's breeding season and probably prevents many individuals from multiple brooding. When this constraint is removed, selection may rapidly favour late nesting.

## 2. Introduction

Blood-feeding ectoparasites often exert strong selective pressure on their hosts and may affect the evolution of life-history traits such as clutch size, immune investment and offspring quality/quantity trade-offs [1–5]. A number of studies have demonstrated negative effects of ectoparasites on nestling growth and survival, especially in colonial hosts where grouping enhances parasite transmission [6–11]. Ectoparasitism may also affect the number of nesting attempts hosts are able to undertake, particularly if infestations increase seasonally to the extent that breeding later—when more parasites are present—is often unsuccessful [12,13]. Ectoparasitism thus could be one of the ecological drivers for the commonly observed seasonal decline in reproductive success for many birds of temperate latitudes [14–18] and might serve to truncate the typical length of the breeding season.

Surprisingly, little is known about how ectoparasites directly affect avian breeding phenology [1]. One study showed that decisions to undertake second broods depended on the extent of parasitism at a site, with birds at lightly infested sites more likely to nest again late in the season [12]. Ectoparasite loads can also impair the productivity of second broods more than that of first broods, with this cost of parasitism increasing over the breeding season [19]. However, because both the decision to nest a second time and the success of late nestings can be affected by a number of environmental factors [16,17,20], determining the role of ectoparasites independent of other effects can be difficult without experimental manipulation of parasite abundance.

In this study, we take advantage of a long-term experiment, in which we annually removed ectoparasitic swallow bugs (Hemiptera: Cimicidae: *Oeciacus vicarius*) from cliff swallow colonies, to investigate the potential role of ectoparasitism in regulating the length of the breeding season. These deleterious blood-feeding parasites are known to affect many aspects of the cliff swallow's social behaviour and ecology [7,8,21,22]. Bugs increase at swallow colony sites over the summer, and thus the seasonally increasing cost of parasitism might prevent birds from undertaking multiple nesting attempts at infested sites. If swallow bugs represent a seasonal constraint on nesting time in cliff swallows, we predicted that cliff swallows should lengthen the time spent breeding at sites where bugs were removed.

Our measure of breeding-season length was whether cliff swallows initiated late nesting at a colony site. Late nesting was defined as a distinct second round of nesting activity (building nests and laying eggs) at a colony site after nestlings had fledged there, generally leading to a doubling of the length of time birds were present at a site. We investigated how the frequency of late nesting changed over a period of more than 20 years at a parasite-free site and use the results to gain insight into how ectoparasites potentially constrain the length of the cliff swallow's breeding season and the number of breeding attempts it makes.

## 3. Material and methods

### 3.1 Study area

We have studied cliff swallows since 1982 in the western Great Plains, USA, centred near the Cedar Point Biological Station (41^°^13′ N, 101^°^39′ W) in Keith County, southwestern Nebraska, along the North and South Platte rivers and including portions of Deuel, Garden, Lincoln and Morrill counties [8]. Cliff swallows construct gourd-shaped mud nests, often in dense, synchronously breeding colonies. In our study area, the birds nest mostly on the sides of bridges, in box-shaped road culverts, or underneath overhangs on the sides of cliffs [23]. Colony size varies widely; in our study area it ranges from 2 to 6000 nests (mean±s.e., 404±13, *n*=2318 colonies), with some birds nesting solitarily. The typical phenology (in the absence of late nesting) is for cliff swallows to first arrive in southwestern Nebraska in mid to late April, for most birds to have initiated egg laying by early June, and for nestlings to have mostly fledged by mid-July. Cliff swallows spend the winter in northeastern Argentina, Uruguay and southwestern Brazil [24], although the wintering range of our specific population is unknown.

Swallow bugs negatively affect nestling cliff swallow development and survival, lowering nestling body mass, inducing anaemia, killing nestlings prior to fledging and increasing nutritional stress and wing asymmetry among birds that fledge [7,8,13,25,26]. Experiments in colonies of different sizes have shown that the effects of bugs are most severe in the largest colonies, where bugs are also the most numerous [7,8], although these colony-size effects have diminished in recent years (C. R. Brown 2014, unpublished data).

### 3.2 Fumigation and study colonies

Swallow bugs were removed from colonies by lightly misting the outside of all cliff swallow nests and adjacent nesting substrate with a dilute solution of the insecticide Dibrom [7,8]. This chemical works largely as a contact pesticide, although we use the term fumigation to describe parasite removal. Nests were sprayed at 7 to 14 day intervals throughout the nesting season, typically beginning early in the season after birds had initially settled at sites and continuing until nests were no longer active. Dibrom is highly effective against swallow bugs [9], and even a single spraying can greatly reduce the number of bugs at a colony site for the entire season (C. R. Brown 2013, personal observation). Other studies of avian ectoparasites have also used this insecticide [25,27]. Dibrom has little effect on the other common haematophagous ectoparasite of cliff swallows in western Nebraska, the bird flea (Siphontaptera: Ceratophyllidae: *Ceratophyllus celsus*), so by fumigating, we removed only swallow bugs [8].

Fumigation of colonies began in 1984 and continued at some sites through to 2014. Some sites were fumigated only in certain years, depending on the research question that required parasite removal. Two colony sites, Whitetail and Junkyard, were fumigated each year of the study, beginning in 1984 and 1998, respectively. The fumigated sites were all concrete road culverts, and they did not differ in physical attributes from other culvert sites occupied by cliff swallows in the study area. Non-fumigated colony sites were those with no fumigation of any part and were situated on culverts, bridges and buildings [23]. Only sites monitored by us throughout the summer and visited often enough to know if late nesting occurred were included in these analyses. Colonies active from 1982 to 2014 were included.

Throughout this paper, a colony site refers to a physical structure at a particular locale where birds nested, whereas a colony refers to a collection of individuals at a given site [23]. At any one time, a site could contain only one colony, as the colony was a functional definition consisting of all birds occupying a given site at a particular time. Colonies occurring at a given colony site in different years were considered independent units of analysis, because colony size at a site and the birds resident there often varied widely from year to year. However, to control for possible non-independence between years brought about by site heterogeneity, we controlled for colony site by including it as a random effect in mixed models.

### 3.3 Measuring late nesting, colony size and initiation date

We assessed whether late nesting occurred at a colony site each year by observing the birds' presence at the site after the early round of nesting had been completed (known by fledging of young from most nests). When the birds' presence at nests at a site after about 15 July suggested they might be nesting, we checked nests for eggs using a dental mirror and flashlight inserted through a nest's tubular mud entrance. For many colonies, we ruled out any late nesting (without checking nests) whenever all birds had vacated the site by 25 July (by which time most cliff swallows had migrated from the study area). Our definition of late nesting as a temporally distinct second round of breeding at a site meant that all colonies included in our analyses had to have been ones that were active during the early round of nesting in May and June (the typical time when cliff swallows nest in the study area) and thus were available to have a late round of nesting. Late nesting typically occurred in nests that had also been occupied in the early round. The birds in the late round at a site had been there during the early round [28] and thus engaged in true double-brooding. There was generally about a 45 to 50 day difference between the mean egg-laying date for the early round at a site and the mean egg-laying date for the late round.

Annual colony sizes were based on first nesting attempts (the early round) and defined as the number of active nests (ones with at least one egg laid) each year. Colony sizes were determined by direct counts of all active nests, known from viewing nest contents, or by estimation based on nest counts of portions of a colony site or from the number of birds present [8].

At Whitetail only, the number of nests active in the late round of nesting was determined each year by either regularly checking all nests in late July and August or estimating the number of active nests from the number of previously banded adults mist-netted at the site after 15 July. The number of late nests was not available for some years. We did not attempt to estimate the number of nests in the late round at any of the other colony sites, where we simply scored the presence or absence of late nesting.

We determined a given colony's initiation date, by recording when the first cliff swallows were observed at the site that year and remained daily thereafter [29]. We monitored colony sites for arriving birds throughout the nesting season, and if initiation date could not be determined exactly or estimated to the nearest 3 days by virtue of our visit schedule, that colony was not included in initiation-date analyses.

As a measure of the relative earliness of each nesting season across the study area independent of a given site's phenology, we used the annual date on which the first juvenile cliff swallow was captured in a mist net (at any colony) in the course of our extensive mark-recapture programme [30,31] conducted each year of the study (except 1982, 1983 and 2014) at 20–35 colonies annually. We netted virtually daily throughout the season, and thus were likely to detect when the first juveniles fledged at any site.

### 3.4 Measuring body mass

As a measure of the quality and condition of individuals initially settling in different colonies, we used the body mass of cliff swallows captured in mist nets at both fumigated and non-fumigated colony sites throughout the study area. We restricted the analysis only to birds captured relatively early in the season when a given colony was engaged in nest-building and egg laying. We thus standardized our body masses temporally with respect to nesting stage, rather than date per se [8], given the wide variance in when colonies initiate within a nesting season [29]. Birds were captured during the course of extensive mark-recapture work [30,31], primarily in mist nets, and weighed to the nearest 0.5 g using a Pesola scale. Sex of cliff swallows was determined using presence/absence of a brood patch or cloacal protuberance [8]. From 1984 to 2013, we had body-mass measurements for 13 287 birds in fumigated colonies and 29 254 birds in non-fumigated colonies. Masses were averaged for any individual caught more than once per year during the designated nesting stage. Each individual was used only once—the year it was first captured—in these analyses, and the same individuals were thus not represented in multiple years.

### 3.5 Palmer drought severity index

As an overall measure of annual climatic conditions during the study (and thus influences on the cliff swallow's aerial insect food), we used the modified Palmer drought severity index (PDSI) for Nebraska's Climate Division 7 (southwest Nebraska) available from the National Oceanic and Atmospheric Administration (NOAA) at www.ncdc.noaa.gov/cag/time-series/. Division 7 encompasses the centre of our study area, and the regional metrics should be broadly representative of climatic conditions the birds experienced each season. The PDSI is a measure of drought intensity used by NOAA, and it integrates both local temperature and rainfall data into a single index useful in describing soil moisture and extent of runoff [32,33]. Lower values of the PDSI indicate more severe drought. Because cliff swallow colonies are active primarily from May through to July, and thus are most likely to be affected by the climatic environment at that time, we used three-month averages for May–July as determined by NOAA.

### 3.6 Statistical analyses

Logistic mixed models were constructed in SAS [34] using Proc GLIMMIX. This allowed specification of colony site as a random effect, with the categorical-dependent variable being whether a colony site exhibited late nesting. Fixed effects were fumigation status (yes/no, categorical), year, colony size, colony initiation date, date of first juvenile capture and PDSI. Multiple regression of the extent of late nesting each year at Whitetail was performed using Proc GLM in SAS. Body mass of birds in fumigated and non-fumigated colonies was compared with Proc MIXED in SAS, with colony site as a random effect and with fixed effects being whether a site was fumigated, colony size, sex and year.

## 4. Results

Of 1183 non-fumigated colonies, 19 (1.6%) had late nesting activity, compared with 49 (75.4%) of 65 fumigated colonies. Using 636 colonies and controlling for colony site as a random effect, only fumigation status of a colony (yes/no) was a significant predictor of whether late nesting occurred (*F*_1,548_=5.24, *p*=0.022). Colony size (*F*_1,548_=0.03, *p*=0.86), colony initiation date (*F*_1,548_=0.41, *p*=0.52), date of first juvenile capture (*F*_1,548_=0.10, *p*=0.75), year (*F*_1,594_=0.36, *p*=0.55) and annual PDSI (*F*_1,594_=0.26, *p*=0.61) were not significant predictors of the presence or absence of late nesting. When the analysis was re-run with an additional 612 colonies (that did not have known initiation dates or were from 3 years without first juvenile capture dates), the results were similar (for fumigation status, *F*_1,1131_=5.15, *p*=0.023; *p*≥0.55 for all others). Colony size, colony initiation date, first juvenile capture date, year and PDSI also had no significant effect on whether colonies exhibited late nesting when fumigated and non-fumigated colonies were analysed separately (*p*≥0.59 for each).

**Figure 1.**
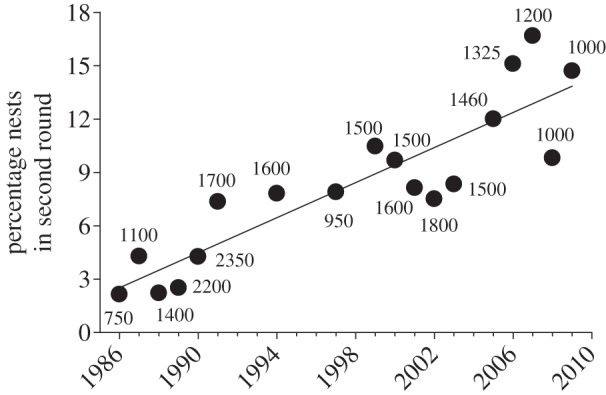
Percentage of cliff swallow nests in the first round of nesting that were active in the second round at a fumigated colony (Whitetail) each year that late nesting occurred. Percentage increased significantly with year (*r*_*s*_=0.89, *p*<0.0001, *n*=18 years). Line indicates best-fit least-squares regression. Numbers by dots indicate colony size (approximate number of nests in first round).

For Whitetail, the percentage of first-round nests that were re-occupied for late nesting increased significantly over time (figure 1), varying from 2.1% in 1986 to a high of 16.7% in 2007. Only year was a significant predictor of the percentage of nests re-occupied for late nesting (*F*_1,15_=31.6, *p*<0.001, *β*=0.49), with Whitetail colony initiation date (*F*_1,15_=0.08, *p*=0.79), annual first juvenile capture date in the study area (*F*_1,15_=0.0, *p*=0.99), Whitetail colony size (*F*_1,15_=0.46, *p*=0.51) and annual PDSI (*F*_1,15_=0.94, *p*=0.35) not explaining any significant variation. Results were similar when colony initiation date and first juvenile capture date were removed from the model.

When controlling for colony site as a random effect, body mass of cliff swallows soon after initial settlement varied significantly with sex (*F*_1,∼42 000_=1621, *p*<0.0001) and year (*F*_27,∼42 000_=76.9, *p*<0.0001) but did not vary with a site's fumigation status (*F*_1,∼42 000_=2.36, *p*=0.12) or with colony size (*F*_1,∼42 000_=0.86, *p*=0.35). Similar results were obtained when each year was analysed separately: in only 2 years (2006, 1997) was fumigation status a significant predictor of body mass (*β*=−1.26 and −1.99, *p*=0.02 and 0.002, respectively), and in the other 25 years fumigation status was not significant (*p*≥0.11 on all). For Whitetail, mean body mass of birds at settlement each year did not change significantly over time (*r*_*s*_=−0.05, *p*=0.82, *n*=26 years).

## 5. Discussion

The extent of ectoparasitism by swallow bugs was clearly associated with the length of the breeding season in cliff swallows of western Nebraska. Colonies with parasite removal via fumigation were over 45 times more likely to have a round of late nesting (and thus a nesting season approximately doubled in length) than were colonies with typical numbers of swallow bugs. Late nesting was unrelated to colony size, phenology of the nesting season or climatic conditions in a given year. That late nesting in Nebraska cliff swallows can be successful and confers potentially higher lifetime fitness than raising only an early single brood [28] suggests that the regular presence of ectoparasites in colonies under natural conditions shortens the birds' nesting season and prevents some individuals from attempting multiple broods.

Our experimental parasite removal establishes the absence of swallow bugs as a causal factor in the initiation of late nesting in cliff swallows. We were able to rule out other possibilities that might have contributed to the observed results. For example, local resources (e.g. the birds' food) could change over time [20], or earlier breeding times in cliff swallows, brought about by responses to increasing drought severity [29], might allow these birds more time in the summer to complete late nesting. However, in either case, we should have seen an effect of year and/or PDSI on the incidence of late nesting at both fumigated and non-fumigated sites, which we did not. Late nesting also seemed unrelated to local phenology (i.e. colony initiation date).

Could the increased late nesting at fumigated sites reflect non-random sorting of high-quality individuals into those colonies? If so, those individuals might have been more capable of nesting late by virtue of their superior condition. The body masses of cliff swallows soon after settlement in colonies do not support this scenario: individual quality or condition, at least as measured by body mass [8], did not differ between fumigated and non-fumigated sites. In addition, colony size itself had no effect on the incidence of late nesting, suggesting that all individuals regardless of their colony-size propensity [35] were equally likely or unlikely to engage in late nesting. Furthermore, because fumigation of a site each year did not begin until after individuals had settled, it is unlikely that individuals had the opportunity to choose their colony (and thus assort) based on whether a site was being fumigated that year. Even at a perennially fumigated site (Whitetail), there was no evidence that the quality of the residents there (as measured by body mass) changed over time as more birds engaged in late nesting.

Cliff swallows' responding to ectoparasites by avoiding late nesting is perhaps not surprising, given the highly deleterious effects of swallow bugs and the other ways cliff swallows adjust their behaviour to bug infestations [7,8,21,25]. Many nests in our study area are so infested with bugs by the time nestlings fledge that neither the adults nor the juveniles ever return to the nests (C. Brown 1984–2014, personal observation), and at these sites the lack of late nesting is not surprising. By contrast, at fumigated sites, parents often lead the juveniles back to the nest to sleep at night for several days after fledging, and the adults themselves (even those who do not attempt late nesting) sit in the empty nests for long periods after the juveniles have permanently vacated the site [36]. In Oklahoma, severe ectoparasite infestations caused entire colonies to abandon nesting sites [13].

While the much greater frequency of late nesting at fumigated colonies compared with non-fumigated ones was striking, perhaps even more surprising was the increase in the extent of late nesting over time at one site (figure 1). This trend could not be explained by changes in breeding phenology, climate or social environment (i.e. colony size). Because late nesting is often perpetrated largely by previous nesters who thus augment their annual reproductive success, the increase over time suggests potential selection for individuals that double-brood. This is supported by brood-size and survival analyses of breeding adults and offspring from early and late nests, which indicate that late nesters have at least equivalent (and perhaps greater) lifetime fitness as those birds that rear only an early brood [28]. However, despite the potential advantages to rearing a second brood, only a relatively small percentage of birds nested late even at fumigated sites such as Whitetail. Other costs of late nesting (e.g. declining food availability, delayed start of autumn migration) independent of ectoparasitism may prevent some individuals from double-brooding, particularly for those who are not among the earliest birds to arrive in the spring [28].

This study demonstrates perhaps the strongest effect yet of a blood-sucking ectoparasite on the length of its host's breeding season and on the host's ability to undertake multiple broods. Our results suggest that parasitism may be an overlooked factor contributing to the wide variability both among and within species in propensity to double-brood in a given year [37–43]. In the case of cliff swallows, their extreme degree of coloniality probably exacerbates the shortened nesting season imposed by parasites because the birds are exposed to such large numbers of swallow bugs by virtue of their large group sizes. This could be one reason why the less social but ecologically similar barn swallow often produces multiple broods in a season in North America [44,45], while the more colonial cliff swallow does not [24]. In geographical areas where swallow bugs are less numerous, multiple brooding should be more common, which appears to be the case [46]. Our experimental results suggest that cliff swallows have a strong degree of plasticity in breeding phenology and that some individuals have the capacity to respond quickly to an absence of parasites.

## Data accessibility

Original data deposited in Dryad, .
